# Commentary: A science-based product regulation: the time has come to reduce toxic emissions to reduce harm

**DOI:** 10.3389/fpubh.2025.1736175

**Published:** 2026-01-12

**Authors:** Mohammad Faisal Wardak

**Affiliations:** 1 Research Center, Ghalib University, Herat, Afghanistan; 2 Paraclinical Department, Faculty of Medicine, Ghalib University, Herat, Afghanistan

**Keywords:** Afghanistan, low- and middle-income countries (LMIC), public health policies, tobacco harm reduction (THR), tobacco regulation, toxic emissions

## Introduction

Kunze, Santin, and Kunze's recent article presents a significant and timely contribution to the ongoing debate on nicotine regulation. By advocating for the classification of nicotine products based on scientifically validated emission thresholds, the authors provide a pragmatic framework for reducing toxicant exposure and informing evidence-based public health policy (1). However, for doctors and public health workers in low-income and fragile health systems like Afghanistan, turning these ideas into real action comes with many practical challenges and ethical questions that deserve careful attention.

## Contextual barriers in low-resource settings

In many low- and middle-income countries (LMICs), tobacco control systems remain weak and unevenly implemented. Afghanistan illustrates these challenges. According to the WHO 2023 Afghanistan Country Profile, 26.2 percent of adults use tobacco, including 45 percent of men and 5.9 percent of women, based on the 2019 national STEPS survey. The report further indicates that 19.3 percent of adults use smokeless tobacco such as naswar, reflecting its deep social and cultural acceptance. While Afghanistan has ratified the WHO Framework Convention on Tobacco Control, the implementation of its key provisions remains limited. National smoke-free laws exist but are poorly enforced, there is no national quit line, and cessation medications and nicotine replacement therapies are not available for legal sale. Health warnings on cigarette packaging are text-only and inconsistently applied, and there have been no recent mass media campaigns to discourage tobacco use (2).

Data from The Tobacco Atlas also show that tobacco kills more than 15,000 Afghans annually and accounts for over 12 percent of all male deaths in the country. The report notes that cigarette affordability has increased since 2008 due to minimal taxation, and tobacco companies continue to advertise freely across retail and informal markets (3). Together, these findings indicate that while Afghanistan possesses some formal policy structures, enforcement mechanisms and cessation support systems are largely absent. Under these conditions, complex emission-threshold regulations are unlikely to be practical or effective. Strengthening enforcement capacity, taxation, and basic cessation services would yield greater public health benefit than implementing laboratory-based regulatory models in such a fragile setting (2, 3).

The emission-threshold regulatory model proposed by Kunze et al. (1) assumes the presence of advanced laboratories, continuous product surveillance, and regulatory mechanisms capable of measuring toxicant output across diverse nicotine products. In most LMICs, however, such resources are scarce and usually limited to a few tertiary centers. Even basic infrastructure such as reliable electricity, trained analytical staff, and quality control systems is often inadequate. As a result, translating emission-based standards into practice remains largely aspirational.

In settings like Afghanistan, where public health budgets are already strained, allocating funds to sophisticated laboratory testing may divert attention from more urgent priorities such as infectious disease control, maternal and child health, and basic tobacco cessation. This imbalance raises critical questions about the ethical use of limited resources. Without strong enforcement systems and sustained funding, implementing high-cost regulatory models risks becoming a symbolic exercise, appearing progressive on paper but yielding little impact in practice.

## Ethical and communication considerations

Kunze et al. (1) propose that science-based regulation can reduce harm by setting emission thresholds for key toxicants. While scientifically valid, its success depends on whether countries can implement it within their social and ethical contexts. In Afghanistan, clinicians often encounter patients wishing to reduce tobacco-related harm but lacking access to cessation support or nicotine replacement therapy. In this setting, Tobacco Harm Reduction (THR) offers a practical and ethically defensible approach. My study, “Tobacco harm reduction in Afghanistan: a recipe for improving smokers' health” (4), found that THR could benefit Afghan smokers if supported by national policy, though barriers such as cost, weak institutional support, and cultural resistance remain.

Similar findings from Pakistan highlight challenges in perception. Media analyses reported THR products like e-cigarettes and nicotine pouches were often portrayed negatively, emphasizing risk and uncertainty over evidence (5). This mirrors Afghanistan, where awareness of relative risk is low and public debate limited.

Without clear, evidence-based communication, emission-threshold regulation could encourage dual use or reinforce safety misconceptions, undermining informed choice and autonomy. For emission-based regulation to achieve the harm-reduction goals of Kunze et al. (1), it must be supported by culturally tailored education, clinician training, and transparent public engagement. Evidence from Afghanistan and Pakistan shows that context-sensitive communication and trust-building are essential (4, 5). Science defines the framework, but its impact depends on people's understanding and acceptance.

## Equity, evidence, and dependency

Most scientific evidence supporting emission-based regulation comes from high-income countries with established laboratory networks and regulatory systems. Directly applying these frameworks to low-income contexts risks widening global inequities in tobacco control. For harm-reduction strategies to be effective worldwide, LMICs must generate their own evidence, validate emission thresholds locally, and build feasible enforcement mechanisms.

Economic considerations are also crucial. Compliance with emission thresholds may favor multinational tobacco companies able to meet costly certification and testing requirements, while smaller local producers could be excluded. This imbalance could limit access to affordable harm-reduction options and undermine public trust. Achieving equity therefore requires science-based regulation to be complemented by policies promoting inclusion, affordability, and fair market participation.

## Conclusion

Kunze et al. (1) highlight the need for science-based regulation to reduce toxic emissions from tobacco and nicotine products. For such frameworks to have public health impact, they must reflect the realities of low-income and fragile health systems. In countries like Afghanistan, limited laboratory infrastructure, regulatory oversight, and cessation services make the challenge technical, ethical, and communicative.

Science-based regulation succeeds only when paired with context-based implementation. Local evidence, equitable policy design, and culturally informed communication are essential to ensure emission-threshold strategies reduce harm without reproducing inequities. As Afghanistan's experience (4) and recent evidence from Pakistan (5) show, tobacco harm reduction depends as much on trust, education, and inclusion as on technology and regulation. Meaningful global progress in harm reduction requires an integrated approach combining science, ethics, and equity to create sustainable and socially legitimate pathways for reducing tobacco-related harm.
